# Literary Notes

**Published:** 1904-11

**Authors:** 


### LITERARY NOTES.

ANNOUNCEMENT is made of the consolidation of *Southern Medicine* and *Gaillard's Medical Journal,* beginning with the October issue. Dr. William Edwards Fitch who established *Southern Medicine* in 1897, under the name of the *Georgia Journal of Medicine and Surgery,* and who has so ably conducted the magazine since that time, will be the editor of the amalgamated publication.

THE *Journal of Obstetrics and Gynecology of the British Empire* is the title of a handsome magazine now in the sixth year of its life. It is edited by Dr. T. W. Eden, assistant obstetric physician at the Charing Cross Hospital, London, with the aid in special departments of D. Berry Hart, Edinburgh; Frederick William Kidd, Dublin, and William Japp Sinclair, of Manchester. With these are associated as an editorial committee Henry Briggs, Liverpool; Murdoch Cameron, Glasgow; Francis Henry Champneys, Charles J. Cullingworth, Alban Doran, George Ernest Herman, and Sir John Williams, London; Edward Malins, Birmingham; Alexander R. Simpson, Edinburgh, and Alfred Smith, Dublin. With these, again, appears a long list of collaborators comprising some of the most prominent physicians in Great Britain and the colonies.

The contents of the journal for October embraces original communications, critical review, review of current literature, review of recent books, and an index obstetricus. It is one of the best special medical magazines published in the English language, and should find its way to the library table of every teacher of gynecology and obstetrics. It is published by Sheratt & Hughes, at 65 Long Acre, W. C., London, and at the University Press, Manchester. Subscription price, single copies, 2s. 6d. net; annual subscription, 25s. post free; colonies and abroad, 26s. post free.

